# Love Thy Neighbour: Group Properties of Gaping Behaviour in Mussel Aggregations

**DOI:** 10.1371/journal.pone.0047382

**Published:** 2012-10-16

**Authors:** Katy R. Nicastro, Gerardo I. Zardi, Christopher D. McQuaid, Gareth A. Pearson, Ester A. Serrão

**Affiliations:** 1 CCMAR-CIMAR Laboratório Associado, Universidade do Algarve, Faro, Portugal; 2 Department of Zoology & Entomology, Rhodes University, Grahamstown, South Africa; University of Otago, New Zealand

## Abstract

By associating closely with others to form a group, an animal can benefit from a number of advantages including reduced risk of predation, amelioration of environmental conditions, and increased reproductive success, but at the price of reduced resources. Although made up of individual members, an aggregation often displays novel effects that do not manifest at the level of the individual organism. Here we show that very simple behaviour in intertidal mussels shows new effects in dense aggregations but not in isolated individuals. *Perna perna* and *Mytilus galloprovincialis* are gaping (periodic valve movement during emersion) and non-gaping mussels respectively. *P. perna* gaping behaviour had no effect on body temperatures of isolated individuals, while it led to increased humidity and decreased temperatures in dense groups (beds). Gaping resulted in cooler body temperatures for *P. perna* than *M. galloprovincialis* when in aggregations, while solitary individuals exhibited the highest temperatures. Gradients of increasing body temperature were detected from the center to edges of beds, but *M. galloprovincialis* at the edge had the same temperature as isolated individuals. Furthermore, a field study showed that during periods of severe heat stress, mortality rates of mussels within beds of the gaping *P. perna* were lower than those of isolated individuals or within beds of *M. galloprovincialis*, highlighting the determinant role of gaping on fitness and group functioning. We demonstrate that new effects of very simple individual behaviour lead to amelioration of abiotic conditions at the aggregation level and that these effects increase mussel resistance to thermal stress.

## Introduction

Evidence across a range of taxa describes animal aggregations as an evolutionarily advantageous strategy that provides individual members with benefits such as reduced predation risk, protection from environmental conditions and increased reproductive success, but at the cost of increased intraspecific competition for resources (e.g. [1]). Although composed of individual members, an aggregation often behaves as an integrated whole and the effect or even the function of a given individual behaviour can be very different at the group level [2], [3]. For example, high temperatures are reached in beehives because of heat produced as a by-product of flying [4]. Intertidal, sedentary animals are commonly assumed to be comparable to plant communities that modify their environment primarily through their structure The physical organization of forest and crop systems strongly modifies environmental factors such as wind speed, solar radiation and relative humidity (e.g. [5]) and similarly for intertidal mussels, the morphological features of individuals and interactions with neighbours can significantly affect their environment. The shielding and shading effects of neighbours alters both hydrodynamic forces and the convective regime, resulting in modified microclimates. Consequently, when subjected to extreme environmental conditions, mussels living in beds are predicted to suffer lower mortality rates than those living as isolated individuals [6]–[8]. However, several recent studies have shown that even very simple behaviour can introduce levels of complexity that cannot be found in plant communities. For example, intertidal mussels, which are sedentary rather than sessile, display a range of individual behaviours that, even if carried out over comparatively long time periods and at small spatial scales, can be important in regulating population dynamics given the extreme spatial and temporal heterogeneity of intertidal habitats [9], [10]. Mussels anchor themselves to the substratum by means of byssal threads, reacting to temporal and environmental changes by adjusting the strength and position of byssal attachment (e.g. [7], [11], [12]). Also, mussels respond to increased availability of space, decreased attachment to neighbours and risk of predation by increasing their movement to re-organize themselves in safer positions within beds (e.g. [13], [14]). Under homogeneous conditions, mussels movement leads to the formation of regularly patterned beds, and it has been shown that pattern formation affects ecosystem-level processes in terms of improved growth and resistance to wave action [15].

As the interface between marine and terrestrial environments, the rocky intertidal zone is an appealing model system to study ecological principles [16], [17], in part because organisms are subjected to sharp gradients in physiological stress and because organisms living in this habitat have been shown to exist at or near the edges of their tolerance limits (e.g. [18], [19]). For many rocky intertidal species, upper vertical limits are thought to be set by physical stresses related to air exposure (i.e. high body temperature or desiccation; e.g. [17], [20], [21]). For example, temperature extremes experienced by mussels during low tide generally far exceed those experienced during submersion [6]. As a response to emersion, some mussel species close the two valves of their shell and undergo anaerobic metabolism, while others exhibit alternate closure and opening of the shell (gaping), allowing the maintenance of aerobic respiration [22], [23]. The first behaviour leads to reduced evaporative water loss at the cost of inefficient use of stored energy; gaping is more compatible with optimal functioning of the metabolic machinery, but valve movements push water out of the mantle cavity, increasing levels of water loss and the risk of desiccation [24], [25]. Although mathematical models suggest that even a small amount of evaporation could lead to a significant loss of heat [6], empirical studies with isolated individuals of different mussel species have shown no significant influence of gaping on body temperature [26]–[28]. Therefore, viewed from the individual level, the evolution of gaping behaviour appears to be linked to increasing aerobic respiration rather than to evaporative cooling [28]–[30]. Mussels normally live in dense beds, however, and the possibility of novel functions and effects of gaping in groups (as opposed to individual members) of such sedentary species has never been examined. Here we study the group effects of gaping behaviour by comparing gaping and non-gaping species as isolated individuals and in aggregations.

In South Africa, *Mytilus galloprovincialis* and *Perna perna* are the two dominant mussel species occurring on intertidal rocky shores. They show partial habitat overlap on the lower shore of the south coast (*M. galloprovincialis* in the upper mussel zone, *P. perna* lower down and co-existence in the mid mussel zone), where they occur as occasional solitary individuals or, more commonly, in very dense beds [31]. *M. galloprovincialis* is a non-gaping species while *P. perna* periodically gapes [32]. We hypothesized that, despite not having an effect on the body temperature of isolated individuals, the sum of individual gaping in dense mussel beds modifies the humidity and temperature environment within the bed and produces reduced body temperatures. Furthermore, we hypothesized that microhabitat amelioration and lower body temperatures experienced within beds of gaping mussels increase resistance to heat stress. Specifically, we tested the following hypotheses:

1. Gaping behaviour does not have an effect on body temperature of solitary individuals.
2. Solitary mussels will be exposed to higher temperatures and lower humidities than mussels living in beds.
3. Conditions in beds of the non-gaping *M. galloprovincialis* will involve higher temperatures and lower humidities than in beds of the gaping *P. perna*.
4. Under field conditions, body temperatures will be highest for isolated individuals and lower in patches of *P. perna* than in patches of *M. galloprovincialis* at the same tidal height.
5. During periods of intense heat stress, mussels in patches of *P. perna* will suffer lower mortality rates than those of solitary mussels or of individuals surrounded by *M. galloprovincialis*.

## Methods

*Mytilus galloprovincialis* and *Perna perna* co-occur as mixed populations in the mid-mussel zone on the south coast of South Africa [31] and experiments and collections were performed in the mid-mussel zone along this stretch of coast. An additional field experiment was performed in Portugal (where the two species also coexist; [33]) at Vilamoura (37°04′36″N, 8°06′42″W) on the 9^th^ of June 2012 (average air temperature was 23°C while maximum was 30°C, Albufeira weather station).

In laboratory experiments, mussels were collected at Plettenberg Bay (34°05′S: 23°19′E) during April to July 2010. Individuals were allowed to recover for four days in oxygenated seawater under acclimation conditions in a controlled environment chamber (19°C under a 12∶12 h light∶dark regime) before deployment and each trial was conducted with fresh mussels.

The group gaping field experiment was conducted at Plettenberg Bay on the 17^th^ of May 2010 (average air temperature was 22°C while maximum was 32.2°C, Plettenberg Bay weather station), while field mortality measures were performed in Algoa Bay (33°58′47″S, 25°39′30″E) in March 2007.

### Individual gaping

In laboratory experiments, ten mussels for each species of 5 cm (±0.5) shell length were used to determine the effect of gaping on body temperatures of single mussels. Five mussels of each species were free to gape and five were prevented from gaping using rubber bands. All mussels were arranged in a circle and oriented parallel to the substratum (one valve facing the substratum the other facing up), and were equidistant from each other (approximately 10 cm). A small hole (<1 mm) was drilled into the posterior end of the mussel shell, and a fine copper/constantan thermocouple wire was inserted into the mantle tissue to measure living mussel body temperature for up to three hours (see [26]).

The experiment was performed at 19 and 25°C (±1°C) and humidity levels of the experimental chambers were maintained at 60% throughout the experiments. Two replicate trials were conducted for each temperature. Throughout each trial, air temperature was monitored. No mortality was observed due to the drilled hole.

#### Statistical analyses

Data fulfilled the pre-requisites for parametric analysis (Shapiro-Wilk test and Cochran's Test) and were analysed using three-factor mixed model ANOVA (GMAV5 [34]) with maximum and average body temperatures as dependent factors, species (*P. perna* or *M. galloprovincialis*) and treatment (allowed to gape or not) as fixed factors and replicated trial (one or two) as a nested random factor.

### Group gaping

#### Laboratory experiments

**Figure 1:**
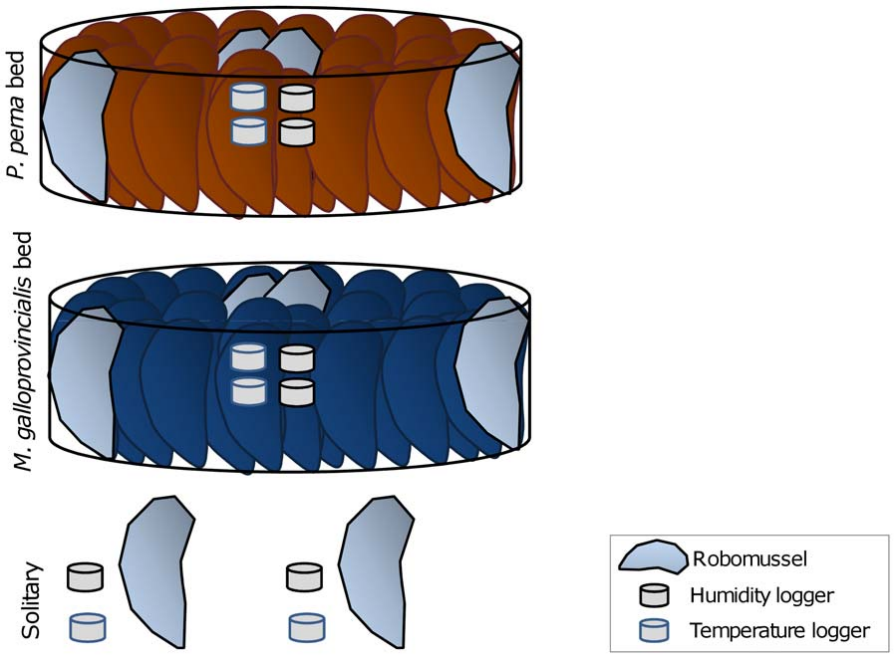
Schematic diagram of the experimental design of the laboratory group experiment. The experiment consisted of three treatments: *P. perna* bed, *M. galloprovincialis* bed and solitary individuals. Three replicates of each treatment were set up and the whole experiment was performed twice.

Robomussels mimic the thermal characteristics of living mussels (see [35]) and were used in all group gaping experiments to avoid potential disruption of the behaviour caused by wires passing over the beds. They were made by placing a temperature logger (iButtons®, Maxim Integrated Products, Dallas Semiconductor, USA) between two empty mussel valves (length ∼5 cm) filled with silicone sealant and left to dry at air temperature for 48 hours before being employed in trials. For the experiment, three artificial mussel beds were made using live mussels for each species, each bed with two robomussels placed in the center of the bed and two at the edge. The artificial beds were circular (diameter of ∼15 cm) and made up of mussels (5 cm±0.5 in length; n∼55) arranged vertically to mimic their position in natural beds. Partially rigid, white PVC net (mesh size 4 cm) was placed around and under the beds to keep the mussels in position. Three sets of robomussels (each consisting of two robomussels 20 cm apart) were placed outside the beds to mimic mussels in isolated positions (referred to as solitary robomussels; Fig. 1). At the beginning of the experiment, bed and solitary treatments were positioned on tiles and aerially exposed to 35°C and 60% humidity for six hours. All laboratory experiments were conducted in a controlled temperature-humidity walk-in room. The temperature chosen is commonly experienced by intertidal mussels on the South African south coast [36]. An additional set of two temperature and two humidity loggers (iButtons®, Maxim Integrated Products, USA) were placed among the mussels at the center of the bed and next to the solitary robomussels for the duration of the experiment.

Two replicate trials were conducted and beds were made 48 hours before the beginning of the experiment to allow further acclimation.

#### Field experiments

Firstly, for each species, four monospecific patches (approx. 20×20 cm) were identified within natural mussel beds. Two robomussels were inserted into the center of each tightly packed bed. At the same location and tidal height, eight solitary robomussels were placed in four patches of bare rock (each of approximately 40×40 cm, two per patch) created by removing mussels and debris around the beds. An additional set of three humidity loggers were located at the center of the bed (amongst mussels) and next to the solitary robomussels for the duration of the experiment.

Additionally, to test if species-specific characteristics other than gaping behaviour affected mussel bed microclimates, a further field experiment was performed. For each species, six artificial mussel beds were made using live mussels and constructed as in laboratory experiments. For each species, three of these beds were made up of individuals free to gape and three with mussels prevented from gaping using rubber bands. Each bed had two robomussels and two ambient and humidity loggers placed in the center.

Beds were made 48 hours before the beginning of the experiment to allow further acclimation and were deployed in the field during a hot sunny summer day, on bare rocky shores in the mid-mussel area and orientated perpendicularly to incoming tide.

#### Statistical analyses

Multiple loggers or multiple robomussels used in each solitary position or each mussel bed were averaged to produce a single datum point. Data fulfilled the pre-requisites for parametric analysis (Shapiro-Wilk test and Cochran's Test) and were analysed using GMAV5 software [34]. Loggers and robomussels recorded data every 3 minutes and maximum (minimum for humidity) and mean values were dependent factors. Post-hoc comparisons involved SNK tests.

For laboratory data, humidity, ambient temperature and robomussel temperatures were analysed using a two-factor mixed model ANOVA with treatment (*M. galloprovincialis* bed, *P. perna* bed, solitary) and replicated trial (one, two) as fixed and nested random factors respectively. For robomussel data, the treatment factor included position in the bed. Pre-treatment differences were tested by analysing time zero data.

For field data, humidity and robomussel temperatures were analysed using one-factor ANOVA with treatment (*M. galloprovincialis* bed, *P. perna* bed, solitary) as a fixed factor. For the additional field experiment, humidity, ambient and robomussel temperatures were analysed using 1-factor ANOVA with bed treatment (*M. galloprovincialis* allowed-to-gape, *P. perna* allowed-to-gape, *M. galloprovincialis* rubber-banded, *P. perna* rubber-banded) as a fixed factor.

### Field mortality

The experiment was designed to test for spatial and seasonal differences in mussel mortality rates, it included several locations on the south coast of South Africa and lasted for one year [12]. The digital pictures of the hottest month taken at one of the experimental sites have been used here to examine the effect of gaping behaviour on mussel mortality during high levels of heat stress.

Digital pictures of 12 quadrats (20×20 cm) were taken and, in each quadrat, 10 individuals of each of the following six categories were selected: individuals of each species surrounded by conspecific mussels, individuals surrounded by individuals of the other species, and solitary individuals of each species. The following month, the same quadrats were photographed and mortality was estimated as disappearance or death of identified individuals between consecutive photographs.

This one month period was chosen as it covered a period of low hydrodynamic and sand stress [12]. In contrast, thermal stress during air exposure was relatively high, with peaks of 37°C, as recorded by robomussel used in Zardi et al. [36] in a nearby (<10 km) location (Cape Recife; 34°01′53″S, 25°42′07″E).

#### Statistical analyses

Data fulfilled the pre-requisites for parametric analysis (Shapiro-Wilk test and Cochran's Test) and were analysed using GMAV5 software [34]. Percentage mortality rates were analysed using one-factor model I ANOVA with treatment (*M. galloprovincialis* in a *M. galloprovincialis* bed, *P. perna* in a *P. perna* bed, *M. galloprovincialis* in a *P. perna* bed, *P. perna* in a *M. galloprovincialis* bed, solitary *P. perna*, solitary *Mytilus galloprovincialis*) as a fixed factor.

## Results

### Individual gaping

**Figure 2:**
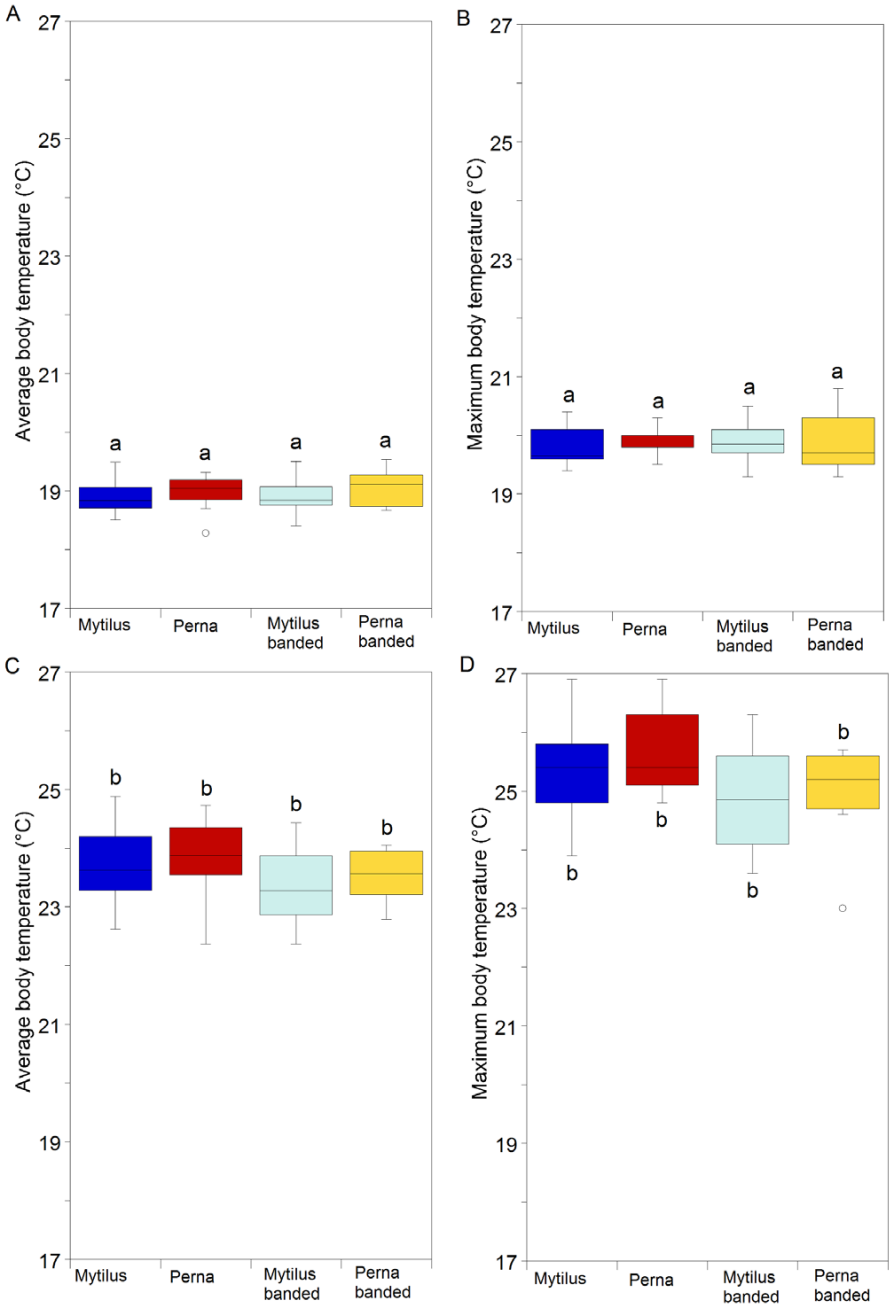
Results of solitary gaping experiments (trials pooled). Average temperatures of mussels at (a) 19°C or (b) 25°C and maximum temperatures of mussels at (c) 19°C or (d) at 25°C. Post hoc results are included in the figure.

Only *Perna perna* gaped at either temperature. Body temperature increased when the experimental chamber was set at the higher temperature (Fig. 2; df = 1, p<0.001, see supporting information Table S1, Table S2), but gaping had no effect on maximum or average body temperatures (df = 1, p>0.05 in all cases). No pre-treatment effects were detected.

### Group gaping

#### Laboratory experiments

At the beginning of the experiments, treatment had no effect on humidity, ambient or robomussel temperatures (data not shown). Humidity, ambient temperature and body temperature recorded by loggers changed throughout the experiment.

**Figure 3:**
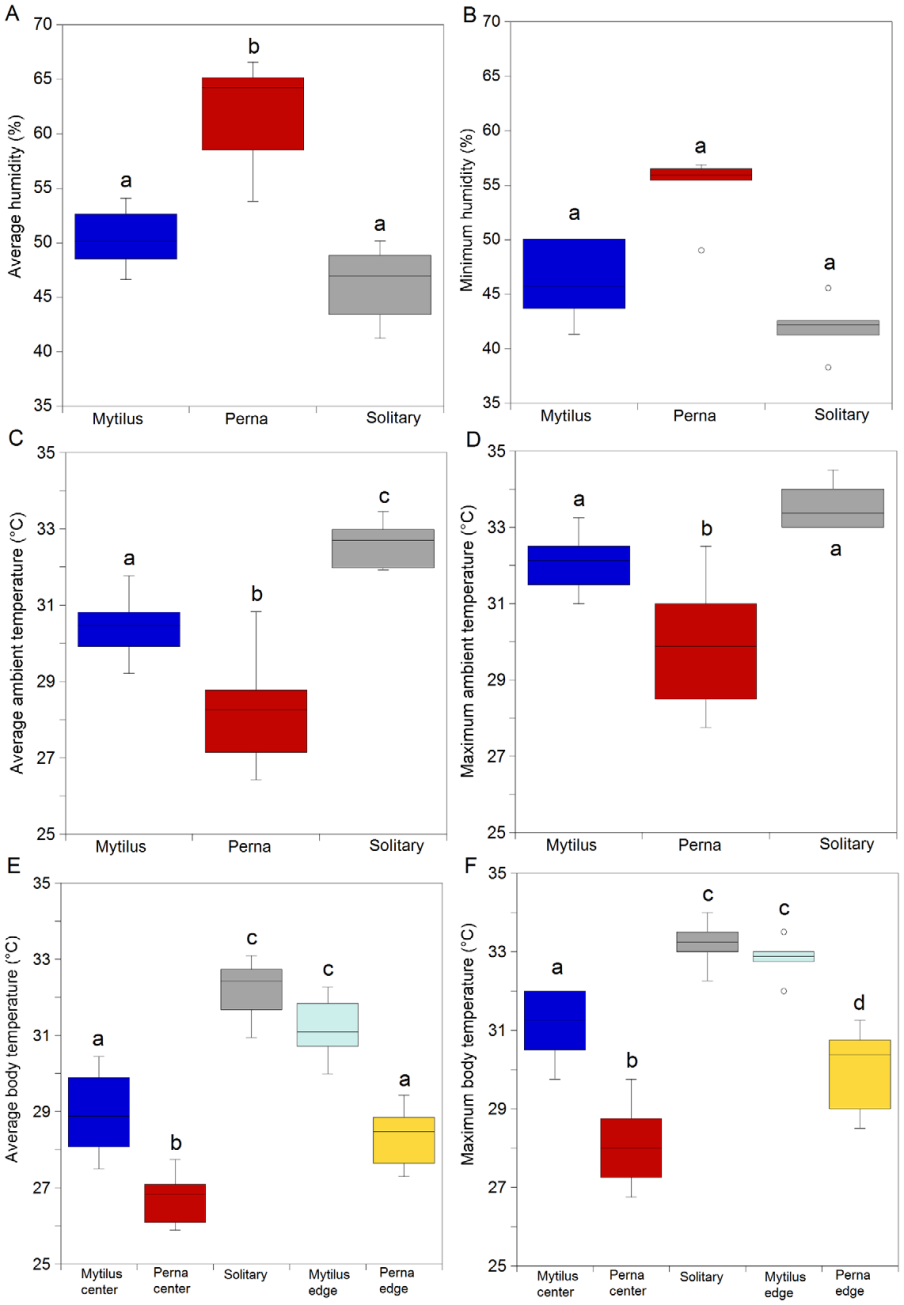
Results of group gaping laboratory experiments (trials pooled). (a) Average and (b) maximum value of humidity; (c) average and (d) maximum value of ambient temperature; (e) average and (f) maximum robomussel temperature. Post hoc results are included in the figure.

ANOVA and SNK tests showed that minimum and average humidity declined significantly in the order *P. perna* beds>*M. galloprovincialis* beds but with no significant difference between *M. galloprovincialis* and solitary mussels. (Fig. 3a, b; p<0.05; Table S3, Table S4). A significant difference in minimum humidity between trials was detected [Trial (Treatment); p<0.05].

Maximum and average body temperatures, as well as ambient values showed a significant treatment effect (Fig. 3c, d; p<0.001; Table S5, Table S6), with values declining in the order *P. perna*<*M. galloprovincialis*<solitary, though *M. galloprovincialis* = solitary for maximum values.

Maximum and average body (robomussel) temperatures showed a gradient of *P. perna* center<*P. perna* edge = *M. galloprovincialis* center<*M. galloprovincialis* edge = solitary (Fig. 3c, f; p<0.001; Table S7, Table S8). Maximum body temperatures also differed as *P. perna* edge<*M. galloprovincialis* center.

#### Field experiments

**Figure 4:**
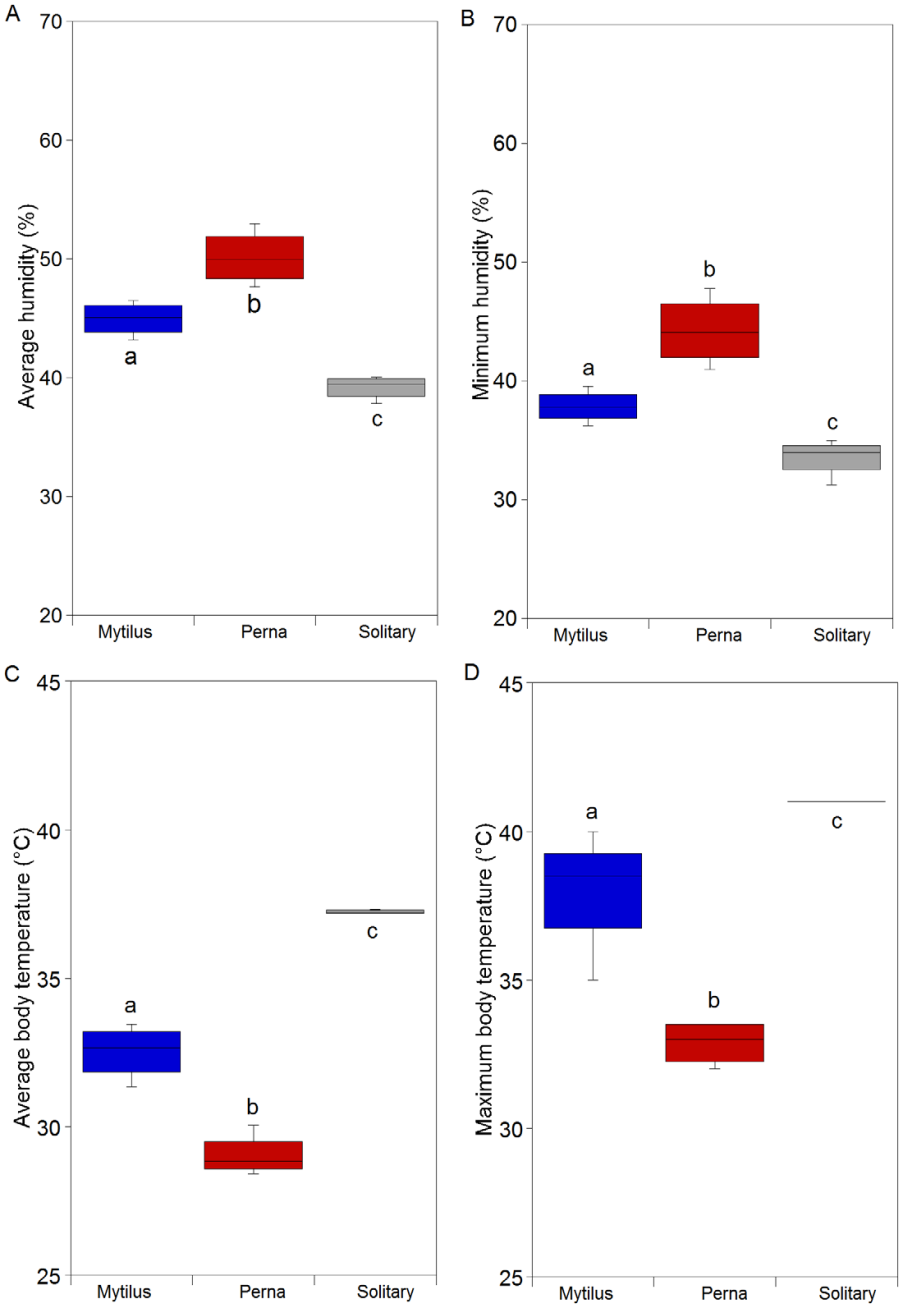
Results of group gaping experiments in the field. (a) Average and (b) maximum value of humidity; (c) average and (d) maximum value of robomussel temperature. Post hoc results are included in the figure. Post hoc results are included in the figure.

Minimum and mean humidity differed significantly among treatments (Fig. 4a, b; p<0.001; Table S9, Table S10): solitary<*M. galloprovincialis*<*P. perna*. Maximum and mean body temperatures showed the reverse: *P. perna*<*M. galloprovincialis*<solitary (Fig. 4c, d; p<0.001; Tables S11, Table S12).

**Figure 5:**
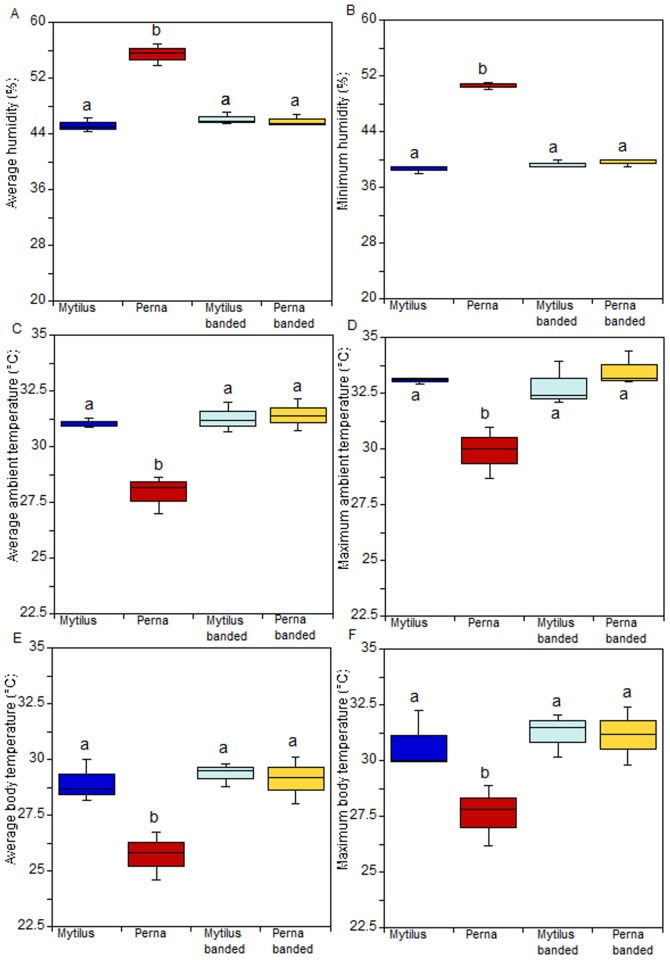
Results of additional group gaping experiment in the field. (a) Average and (b) maximum value of humidity; (c) average and (d) maximum value of ambient temperature; (e) average and (f) maximum robomussel temperature. Banded refers to mussels artificially prevented from gaping, others were free to gape. Post hoc results are included in the figure.

In the additional experiment, humidity values, as well as ambient and body temperatures, showed a significant treatment effect (Fig. 5a,b,c; p<0.001; Table S13, Table S14, Table S15, Table S16, Table S17, Table S18) because values for the *P. perna* treatment in which individuals were allowed to gape differed significantly from all other treatments.

### Field mortality

**Figure 6:**
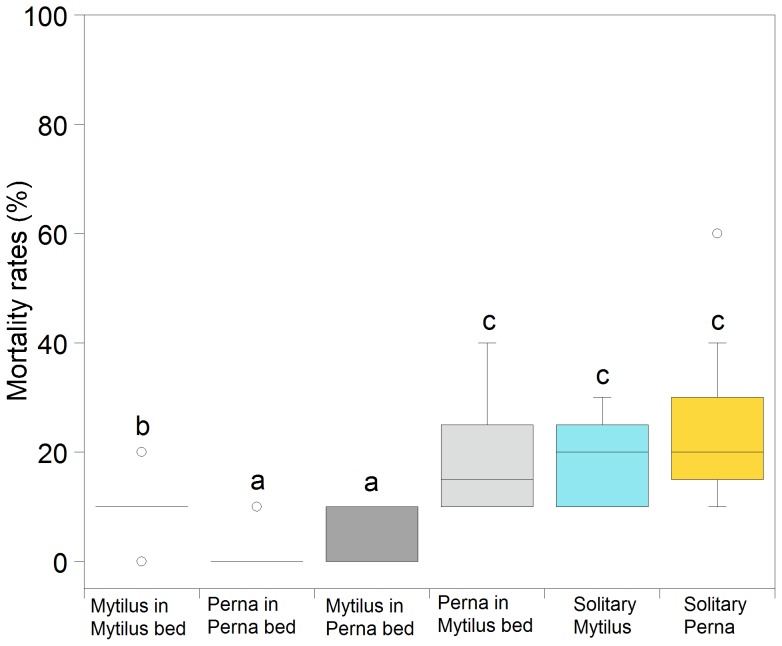
Results of field mortality experiment. Percentage mortality rates measured in the field after a month of high thermal stress and low wave and sand stress. Post hoc results are included in the figure.

Percentage mortality differed significantly among treatments: *P. perna* in a *P. perna* bed = *M. galloprovincialis* in a *P. perna* bed<*M. galloprovincialis* in a *M. galloprovincialis* bed<*P. perna* in a *M. galloprovincialis* bed = solitary *Mytilus galloprovincialis* = solitary *P. perna* (Fig. 6; arcsine transformation; p<0.001; Table S19).

## Discussion

We show that, despite not having an effect on body temperature of isolated individuals, gaping behaviour in mussel beds can significantly modify the habitat and thermal environment experienced by individual organisms composing the aggregation. Furthermore, a field study showed that, during periods of intense heat stress, survival rates of mussels surrounded by gaping mussels are higher than those of isolated individuals or amongst non-gaping mussels.

Several recent studies indicate that even very simple behaviours of sedentary taxa can have profound effects, contributing to effective habitat partitioning, survival and the outcome of interactions between co-existing species [14], [15], [37]. Gaping by mussels allows aerobic respiration by maintaining an oxygen gradient across the gills and the mantle wall, but has no cooling effect on body temperature of isolated individuals [26], [28], [30] and we confirm this for *Perna perna*. However, mussels normally occur in dense aggregations and our field and laboratory experiments revealed an additional ecological role of gaping behaviour that manifests in mussel aggregations but not in solitary animals. The synergistic effect of gaping behaviour in aggregation strongly modifies thermal and humidity conditions. Aggregation in the non-gaping *Mytilus galloprovincialis* leads to lower air and body temperatures and higher humidity levels than experienced by solitary mussels, but this effect was significantly enhanced by combined gaping in *P. perna* beds probably through the evaporation of water discharged during valve movements. In fact, gaping behaviour increases water loss [32]. When aerially exposed, *P. perna* loses more water than the non-gaping species, due to both evaporation and expulsion of water during valve closure. After six hours air exposed at 37°C *P. perna* and *M. galloprovincialis* lose an average of 46% and 16% of total body water respectively [32], demonstrating that gaping affects tolerance to desiccation and possibly influences the vertical zonation of the two species. Non-gaping behavior allows the invasive species to colonise the higher mussel zone where the risk of desiccation is high. On the other hand, *P. perna* is restricted by its gaping behavior to the lower shore where it is also able to exclude the other species [32]. Moreover, respiration rates of organisms usually increase with temperature and several studies have shown that mussel oxygen consumption increases exponentially with temperature (e. g. [23]). In *P. perna*, percentage of gaping individuals and ventilation movements (number of gapes per hour) increase at high temperatures [32]. Specifically, gaping is observed for only the first two hours at low temperatures (17°C), while about 40% of mussels keep gaping for longer at higher temperatures (37°C). Most probably the correlation between gaping and heat stress results from greater oxygen consumption when exposed in air to higher temperatures.

Classically, aggregation is considered as an evolutionarily advantageous condition, in which members gain the benefits of protection, mate choice, and centralized information, at the price of limiting resources [1]. Certain environmental factors, and their associated risks, can lead to a direct increase in the aggregation response and/or indirectly increase benefits associated with living in aggregations. A common environmental risk is thermal stress (e.g. [38]–[40]): whether ultimately a cause or a consequence of group living, the body temperature of solitary and gregarious animals is likely to differ and may thus have important ecological consequences, potentially affecting species phenologies, microhabitat use, distributions, and responses to climate change. Thermoregulation through aggregation occurs across a wide range of taxa and habitats including birds [41] and terrestrial snails [42], but is likely to be particularly relevant in intertidal ecosystems where many organisms are thought to live close to their thermal limits [18], [19], [21]. Thermal tolerance can control local and latitudinal biogeographic patterning of many intertidal species [36], [43], [44], and there is increasing evidence that micro-scale thermal variability can surpass large-scale gradients (e.g. [45], [46]). Which effects in groups are biologically significant and which are simply epiphenomena that are not necessarily evolutionarily important is still a fundamental question. Our results show that, during periods of particularly intense heat conditions, mortality rates of mussels surrounded by gaping *P. perna* individuals were significantly lower than those of solitary individuals (of either species) or mussels surrounded by non-gaping *M. galloprovincialis*. This indicates that gaping affects ecosystem-level processes in terms of improved resistance to heat stress, suggesting an evolutionary significance for changes in temperature and humidity induced by this behaviour. It is important to note that on the south coast of South Africa, hydrodynamic stress, together with high air temperatures, is considered the most important stress factor affecting mussel mortality. The survey of this study was conducted at sheltered in-bay locations and during periods of minimal wave action [12], therefore most of mortality can be attributed to thermal stress.

Although gaping behaviour is one obvious difference between *M. galloprovincialis* and *P. perna*, several other attributes may alter intertidal invertebrate body temperatures. For example, thermal mass for a standard length, shell thickness and shape and the projected area that faces towards incoming solar radiation all have strong effects on body temperatures [6]. We clearly show that temperature and humidity within beds of *M. galloprovincialis* (rubber-banded and allowed-to-gape) and within beds of *P. perna* that were prevented from gaping were not significantly different, while they were significantly different for beds in which *P. perna* were free to gape. These results exclude the possibility that species-specific traits, other than gaping behaviour could be responsible for altering temperature and humidity conditions in the mussel bed microhabitat.

Environmental stresses are not experienced equally by all members of a group so that variability in the spatial structure of an aggregation results in different effects on the fitness of each individual [1], [47]. For example, in terrestrial snails, there are clear gradients in body temperature within aggregations that are related to shading of the substratum [42] and in mussels, body size, body position in the micro-habitat and position in the aggregation can profoundly modify the temperature each individual experiences [6], [27]. Our results show that in mussel aggregations the effects of gaping behaviour are more evident at the centre of mussel beds. In both species, mussel body temperature is lower in the centre of beds than at the edges. At the end of the experiments, mussels at the edge of beds of non-gaping species had the same body temperatures as solitary individuals, while this was not true for individuals at the edge of *P. perna* beds. We considered only monolayered beds, while in nature mussels often form multilayered beds [48], [49]. These are very dense and structurally complex matrices made of several strata of mussels also common at the experimental sites used in this study (personal observations KN and GIZ). Future studies will help to understand if the gaping effects we observed in monolayered beds are even more pronounced in intricate, crowded multilayered beds.

By interacting with the diverse biological attributes of species, environmental heterogeneity plays a defining role in invasion and coexistence dynamics [50]–[52]. As both indigenous species and invaders respond to environmental variations, it is the difference in their responses that determines the success of the invader and how it interacts with native species [53], [54]. The Mediterranean mussel *M. galloprovincialis* is one of the most widespread marine invasive species worldwide and it is the most successful marine invader in South Africa [55]. Along the south coast, it shows partial habitat segregation with indigenous *P. perna* resulting from a combination of competitive effects and differing physiological capacities along gradients of multiple stressors [8], [56]. It has been recognized that facilitation (positive species interactions) can be as biologically significant as other factors (e.g. competition, predation, physical stress) in a variety of environments driving rapid evolutionary changes and influencing the dynamics of populations and communities [57]–[59]. Previous studies have shown that because of its higher attachment strength, *P. perna* initially facilitates survival of the invasive *M. galloprovincialis* by providing protection against waves [12], [60]. Our results offer novel insights into the interaction dynamics between these two species and more generally indicate that behavioural properties of an indigenous species have the potential to facilitate invasion by mitigating harsh environmental regimes. In addition, we show that, *P. perna* individuals within *M. galloprovincialis* beds experienced mortality rates similar to those of solitary individuals. This result is in accordance with previous studies highlighting the costs of high desiccation rates of individual gaping mussels when facing high temperature and desiccation stress [32].The beneficial effect of this behaviour appears be evident at the group level, but it is absent or minimal in solitary positions or among non-gaping species.

In this study, we exploited contrasting behaviours of coexisting intertidal mussel species to highlight the novel effects of group gaping as opposed to individual gaping. We confirmed our initial hypotheses and showed that gaping behaviour can change the proximal microhabitat to moderate an organism's experience of environmental conditions radically, but that this occurs only in mussel aggregations. Furthermore, lower mortality rates experienced by individuals in the middle of gaping mussels during a period of high thermal stress highlights the evolutionary relevance of gaping-induced small scale facilitation for the structure and functioning of mussel beds. Finally, we stress the importance of observing behavioural patterns at organizational levels that are meaningful to the organism in order to develop a realistic understanding of their ecological significance.

## Supporting Information

Table S1 **Results** **of the ANOVA applied to the maximum body temperatures recorded during the solitary gaping experiments.** Results of the three-factor mixed model ANOVA with maximum body temperatures as dependent factors and with species (*M. galloprovincialis* bed, *P. perna*) and treatment (allowed to gape or not) as a fixed factor and replicated trial (one or two) as a nested random factors.
(DOCX)

Table S2 **Results** **of the ANOVA applied to the average body temperatures recorded during the solitary gaping experiments.** Results of the three-factor mixed model ANOVA with average body temperatures as dependent factors and with species and treatment (allowed to gape or not) as a fixed factor and replicated trial (one or two) as a nested random factors.
(DOCX)

Table S3 **Results** **of the ANOVA applied to the minimum humidity recorded during the group gaping laboratory experiments.** Results of the two-factor mixed model ANOVA with treatment (*M. galloprovincialis* bed, *P. perna* bed, solitary) and replicated trial (one, two) as fixed and nested random factors respectively.
(DOCX)

Table S4 **Results** **of the ANOVA applied to the average humidity recorded during the group gaping laboratory experiments.** Results of the two-factor mixed model ANOVA with treatment (*M. galloprovincialis* bed, *P. perna* bed, solitary) and replicated trial (one, two) as fixed and nested random factors respectively.
(DOCX)

Table S5 **Results** **of the ANOVA applied to the maximum ambient temperatures recorded during the group gaping laboratory experiments.** Results of the two-factor mixed model ANOVA with treatment (*M. galloprovincialis* bed, *P. perna* bed, solitary) and replicated trial (one, two) as fixed and nested random factors respectively.
(DOCX)

Table S6 **Results** **of the ANOVA applied to the average ambient temperatures recorded during the group gaping laboratory experiments.** Results of the two-factor mixed model ANOVA with treatment (*M. galloprovincialis* bed, *P. perna* bed, solitary) and replicated trial (one, two) as fixed and nested random factors respectively.
(DOCX)

Table S7 **Results** **of the ANOVA applied to the maximum body (robomussel) temperatures recorded during the group gaping laboratory experiments.** Results of the two-factor mixed model ANOVA with treatment (*M. galloprovincialis* bed, *P. perna* bed, *M. galloprovincialis* edge, *P. perna* edge, solitary) and replicated trial (one, two) as fixed and nested random factors respectively.
(DOCX)

Table S8 **Results** **of the ANOVA applied to the average body (robomussel) temperatures recorded during the group gaping laboratory experiments.** Results of the two-factor mixed model ANOVA with treatment (*M. galloprovincialis* bed, *P. perna* bed, *M. galloprovincialis* edge, *P. perna* edge, solitary) and replicated trial (one, two) as fixed and nested random factors respectively.
(DOCX)

Table S9 **Results** **of the ANOVA applied to the minimum humidity recorded during the group gaping field experiments.** Results of the one-factor model ANOVA with treatment (*M. galloprovincialis* bed, *P. perna* bed, solitary) as a fixed factor.
(DOCX)

Table S10 **Results** **of the ANOVA applied to the average humidity recorded during the group gaping field experiments.** Results of the one-factor model ANOVA with treatment (*M. galloprovincialis* bed, *P. perna* bed, solitary) as a fixed factor.
(DOCX)

Table S11 **Results** **of the ANOVA applied to the maximum body (robomussel) temperatures recorded during the group gaping field experiments.** Results of the one-factor model ANOVA with treatment (*M. galloprovincialis* bed, *P. perna* bed, solitary) as a fixed factor.
(DOCX)

Table S12 **Results** **of the ANOVA applied to the average body (robomussel) temperatures recorded during the group gaping field experiments.** Results of the one-factor model ANOVA with treatment (*M. galloprovincialis* bed, *P. perna* bed, solitary) as a fixed factor.
(DOCX)

Table S13 **Results** **of the ANOVA applied to the minimum humidity recorded during the additional group gaping field experiments.** Results of the one-factor model ANOVA with treatment (*M. galloprovincialis* allowed-to-gape, *P. perna* allowed-to-gape, *M. galloprovincialis* rubber-banded, *P. perna* rubber-banded) as a fixed factor.
(DOCX)

Table S14 **Results** **of the ANOVA applied to the average humidity recorded during the additional group gaping field experiments.** Results of the one-factor model ANOVA with treatment (*M. galloprovincialis* allowed-to-gape, *P. perna* allowed-to-gape, *M. galloprovincialis* rubber-banded, *P. perna* rubber-banded) as a fixed factor.
(DOCX)

Table S15 **Results** **of the ANOVA applied to maximum ambient temperatures recorded during the additional group gaping field experiments.** Results of the one-factor model ANOVA with treatment (*M. galloprovincialis* allowed-to-gape, *P. perna* allowed-to-gape, *M. galloprovincialis* rubber-banded, *P. perna* rubber-banded) as a fixed factor.
(DOCX)

Table S16 **Results** **of the ANOVA applied to average ambient temperatures recorded during the additional group gaping field experiments.** Results of the one-factor model ANOVA with treatment (*M. galloprovincialis* allowed-to-gape, *P. perna* allowed-to-gape, *M. galloprovincialis* rubber-banded, *P. perna* rubber-banded) as a fixed factor.
(DOCX)

Table S17 **Results** **of the ANOVA applied to the maximum body (robomussel) temperatures recorded during the additional group gaping field experiments.** Results of the one-factor model ANOVA with treatment (*M. galloprovincialis* allowed-to-gape, *P. perna* allowed-to-gape, *M. galloprovincialis* rubber-banded, *P. perna* rubber-banded) as a fixed factor.
(DOCX)

Table S18 **Results** **of the ANOVA applied to the average body (robomussel) temperatures recorded during the additional group gaping field experiments.** Results of the one-factor model ANOVA with treatment (*M. galloprovincialis* allowed-to-gape, *P. perna* allowed-to-gape, *M. galloprovincialis* rubber-banded, *P. perna* rubber-banded) as a fixed factor.
(DOCX)

Table S19 **Results** **of the ANOVA applied to the percentage of mortality rates.** Results of the one-factor ANOVA with treatment (*M. galloprovincialis* in a *M. galloprovincialis* bed, *P. perna* in a *P. perna* bed, *M. galloprovincialis* in a *P. perna* bed, *P. perna* in a *M. galloprovincialis* bed, solitary *P. perna*, solitary *Mytilus galloprovincialis*) as a fixed factor.
(DOCX)
