# Supplementary material for: Love Thy Neighbour: Group Properties of Gaping Behaviour in Mussel Aggregations
Source: PLoS One. 2012 Oct 16;7(10):e47382. doi: 10.1371/journal.pone.0047382 (PMC3472978; doi:10.1371/journal.pone.0047382)
Supplement: Table S1 — Results of the ANOVA applied to the maximum body temperatures recorded during the solitary gaping experiments. Results of the three-factor mixed model ANOVA with maximum body temperatures as dependent factors and with species (M. galloprovincialis bed, P. perna) and treatment (allowed to gape or not) as a fixed factor and replicated trial (one or two) as a nested random factors. (DOCX) [file pone.0047382.s001.docx]

**Table 1S**

| Source | DF | MS | F | P |
| --- | --- | --- | --- | --- |
| Species | 1 | 0.4914 | 1.57 | 0.2452 |
| Temperature | 1 | 430.9169 | 1379.49 | 0.0001 |
| Treatment | 1 | 0.5561 | 1.78 | 0.2188 |
| Trial (Species X Temperature X Treatment) | 8 | 0.3124 | 1.36 | 0.2329 |
| Species X Temperature | 1 | 0.0256 | 0.08 | 0.7821 |
| Species X Treatment | 1 | 0.0546 | 0.17 | 0.6869 |
| Temperature X Treatment | 1 | 0.7586 | 2.43 | 0.1578 |
| Species X Temperature X Treatment | 1 | 0.0033 | 0.01 | 0.9213 |
| RES | 64 | 0.2303 |  |  |
| TOT | 79 |  |  |  |
