# Supplementary material for: Love Thy Neighbour: Group Properties of Gaping Behaviour in Mussel Aggregations
Source: PLoS One. 2012 Oct 16;7(10):e47382. doi: 10.1371/journal.pone.0047382 (PMC3472978; doi:10.1371/journal.pone.0047382)
Supplement: Table S2 — Results of the ANOVA applied to the average body temperatures recorded during the solitary gaping experiments. Results of the three-factor mixed model ANOVA with average body temperatures as dependent factors and with species and treatment (allowed to gape or not) as a fixed factor and replicated trial (one or two) as a nested random factors. (DOCX) [file pone.0047382.s002.docx]

**Table 2S**

| Source | DF | MS | F | P |
| --- | --- | --- | --- | --- |
| Species | 1 | 0.2880 | 0.51 | 0.4958 |
| Temperature | 1 | 577.8125 | 1021.32 | 0.0001 |
| Treatment | 1 | 1.2500 | 2.21 | 0.1755 |
| Trial (Species X Temperature X Treatment) | 8 | 0.5658 | 1.45 | 0.1943 |
| Species X Temperature | 1 | 0.1805 | 0.32 | 0.5877 |
| Species X Treatment | 1 | 0.0720 | 0.13 | 0.7305 |
| Temperature X Treatment | 1 | 1.7405 | 3.08 | 0.1175 |
| Species X Temperature X Treatment | 1 | 0.0245 | 0.04 | 0.8404 |
| RES | 64 | 0.3908 |  |  |
| TOT | 79 |  |  |  |
