# Supplementary material for: Love Thy Neighbour: Group Properties of Gaping Behaviour in Mussel Aggregations
Source: PLoS One. 2012 Oct 16;7(10):e47382. doi: 10.1371/journal.pone.0047382 (PMC3472978; doi:10.1371/journal.pone.0047382)
Supplement: Table S4 — Results of the ANOVA applied to the average humidity recorded during the group gaping laboratory experiments. Results of the two-factor mixed model ANOVA with treatment (M. galloprovincialis bed, P. perna bed, solitary) and replicated trial (one, two) as fixed and nested random factors respectively. (DOCX) [file pone.0047382.s004.docx]

**Table 4S**

| Source | DF | MS | F | P |
| --- | --- | --- | --- | --- |
| Treatment | 2 | 405.4025 | 34.21 | 0.0086 |
| Trial (Treatment) | 3 | 11.8489 | 0.90 | 0.4694 |
| RES | 12 | 13.1588 |  |  |
| TOT | 17 |  |  |  |
