# Supplementary material for: Love Thy Neighbour: Group Properties of Gaping Behaviour in Mussel Aggregations
Source: PLoS One. 2012 Oct 16;7(10):e47382. doi: 10.1371/journal.pone.0047382 (PMC3472978; doi:10.1371/journal.pone.0047382)
Supplement: Table S8 — Results of the ANOVA applied to the average body (robomussel) temperatures recorded during the group gaping laboratory experiments. Results of the two-factor mixed model ANOVA with treatment (M. galloprovincialis bed, P. perna bed, M. galloprovincialis edge, P. perna edge, solitary) and replicated trial (one, two) as fixed and nested random factors respectively. (DOCX) [file pone.0047382.s008.docx]

**Table 8S**

| Source | DF | MS | F | P |
| --- | --- | --- | --- | --- |
| Treatment | 4 | 29.0317 | 47.11 | 0.0004 |
| Trial (Treatment) | 5 | 0.6162 | 0.82 | 0.5526 |
| RES | 20 | 0.7555 |  |  |
| TOT | 29 |  |  |  |
