# Supplementary material for: Love Thy Neighbour: Group Properties of Gaping Behaviour in Mussel Aggregations
Source: PLoS One. 2012 Oct 16;7(10):e47382. doi: 10.1371/journal.pone.0047382 (PMC3472978; doi:10.1371/journal.pone.0047382)
Supplement: Table S10 — Results of the ANOVA applied to the average humidity recorded during the group gaping field experiments. Results of the one-factor model ANOVA with treatment (M. galloprovincialis bed, P. perna bed, solitary) as a fixed factor. (DOCX) [file pone.0047382.s010.docx]

**Table 10S**

| Source | DF | MS | F | P |
| --- | --- | --- | --- | --- |
| Treatment | 2 | 115.9116 | 26.47 | 0.0002 |
| RES | 9 | 4.3787 |  |  |
| TOT | 11 |  |  |  |
