# Supplementary material for: Love Thy Neighbour: Group Properties of Gaping Behaviour in Mussel Aggregations
Source: PLoS One. 2012 Oct 16;7(10):e47382. doi: 10.1371/journal.pone.0047382 (PMC3472978; doi:10.1371/journal.pone.0047382)
Supplement: Table S13 — Results of the ANOVA applied to the minimum humidity recorded during the additional group gaping field experiments. Results of the one-factor model ANOVA with treatment (M. galloprovincialis allowed-to-gape, P. perna allowed-to-gape, M. galloprovincialis rubber-banded, P. perna rubber-banded) as a fixed factor. (DOCX) [file pone.0047382.s013.docx]

**Table 13S**

| Source | DF | MS | F | P |
| --- | --- | --- | --- | --- |
| Treatment | 3 | 119.0231 | 241.67 | 0.0001 |
| RES | 8 | 0.4925 |  |  |
| TOT | 11 |  |  |  |
