# Supplementary material for: Love Thy Neighbour: Group Properties of Gaping Behaviour in Mussel Aggregations
Source: PLoS One. 2012 Oct 16;7(10):e47382. doi: 10.1371/journal.pone.0047382 (PMC3472978; doi:10.1371/journal.pone.0047382)
Supplement: Table S17 — Results of the ANOVA applied to the maximum body (robomussel) temperatures recorded during the additional group gaping field experiments. Results of the one-factor model ANOVA with treatment (M. galloprovincialis allowed-to-gape, P. perna allowed-to-gape, M. galloprovincialis rubber-banded, P. perna rubber-banded) as a fixed factor. (DOCX) [file pone.0047382.s017.docx]

**Table 17S**

| Source | DF | MS | F | P |
| --- | --- | --- | --- | --- |
| Treatment | 3 | 8.8137 | 5.68 | 0.0222 |
| RES | 8 | 1.5529 |  |  |
| TOT | 11 |  |  |  |
