# Supplementary material for: Love Thy Neighbour: Group Properties of Gaping Behaviour in Mussel Aggregations
Source: PLoS One. 2012 Oct 16;7(10):e47382. doi: 10.1371/journal.pone.0047382 (PMC3472978; doi:10.1371/journal.pone.0047382)
Supplement: Table S19 — Results of the ANOVA applied to the percentage of mortality rates. Results of the one-factor ANOVA with treatment (M. galloprovincialis in a M. galloprovincialis bed, P. perna in a P. perna bed, M. galloprovincialis in a P. perna bed, P. perna in a M. galloprovincialis bed, solitary P. perna, solitary Mytilus galloprovincialis) as a fixed factor. (DOCX) [file pone.0047382.s019.docx]

**Table 19S**

| Source | DF | MS | F | P |
| --- | --- | --- | --- | --- |
| Treatment | 5 | 1578.751 | 27.26 | 0.0001 |
| RES | 66 | 57.9124 |  |  |
| TOT | 71 |  |  |  |
